# Impact of Particle Size on the Aerobic Decomposition and Fertilizer Efficiency of Corn Cobs: A Sustainable Waste-to-Resource Approach

**DOI:** 10.3390/biology14111610

**Published:** 2025-11-17

**Authors:** Qian Liu, Pengbing Wu, Xingchi Guo, Ying Qu, Junyan Zheng, Yuhe Xing, Zhiyu Dong, Wei Yu, Guoyu Zhang, Xu Zhang

**Affiliations:** 1 College of Landscape Architecture, Changchun University, Changchun 130012, China; 2 Institute of Resource Utilization and Soil Conservation, Changchun University, Changchun 130022, China

**Keywords:** corn cob particle size, fertilization efficiency, hydrolysis, microbial community, sustainable

## Abstract

The conversion of agricultural residues into high-value organic amendments is fundamental to sustainable farming systems. Corn cobs represent a widely available lignocellulosic resource; however, their rigid structural properties often hinder efficient biodegradation during composting. This study evaluated whether optimizing corn cob particle size could improve aerobic composting performance by enhancing humification and compost quality. Corn cobs were ground into three particle sizes (6-mesh, 10-mesh, and 20-mesh) and composted with a commercial microbial inoculant for up to 51 days. Physicochemical properties, humic substance fractions (HSC, HAC, FAC), microbial community dynamics (16S rRNA and ITS sequencing), and maturity indicators were monitored. The 10-mesh treatment (M10) exhibited the most favorable composting outcomes, achieving the greatest degree of humification (HA/FA = 2.85; HAC = 48.30 g/kg) and the most pronounced aromatic condensation in humic acids. M10 also supported a more diverse and metabolically specialized microbial consortium, with notable enrichment of lignocellulose-degrading and humus-forming genera (e.g., Streptomyces, Thermobifida). Consequently, M10 produced the most mature compost, reflected by the highest germination index (93.63%) and the lowest heavy-metal accumulation, meeting agricultural safety standards. Structural equation modeling revealed that particle size influenced humification primarily by modulating microbial community structure (path coefficient = 0.86), highlighting particle size as a key environmental selector in composting systems. Overall, 10-mesh particle size created an optimal aeration–moisture balance that stimulated microbial metabolism, accelerated organic matter degradation, and enhanced stable organic matter formation. These findings demonstrate that corn cob particle size significantly governs composting efficiency and final product quality. Selecting a 10-mesh size presents a practical pretreatment strategy to accelerate biomass turnover and produce safe, nutrient-rich compost, providing an effective approach for sustainable bioconversion of agricultural residues.

## Simple Summary

Corn cobs are abundant agricultural residues that can be recycled into compost to improve soil fertility and support sustainable farming. However, their fibrous structure makes them slow to break down, and efficient composting depends on optimizing processing conditions. This study examined how different corn-cob particle sizes affect aerobic composting performance. We found that particle size strongly influenced temperature evolution, organic-matter degradation, nutrient retention, and humification. Medium-sized particles offered the best balance between aeration and moisture, promoting active microbial metabolism, faster decomposition, and higher compost maturity compared with finer or coarser particles. These results show that selecting an appropriate particle size can significantly improve composting efficiency and compost quality when recycling corn residues. This strategy provides a practical and sustainable pathway to convert crop waste into nutrient-rich fertilizer, reduce dependence on synthetic inputs, and enhance soil health in agricultural systems.

## 1. Introduction

Agricultural waste, particularly residues such as corn cobs, presents substantial environmental challenges when improperly managed. In many agricultural regions, the disposal of these residues through open burning or landfilling contributes to air pollution, soil degradation, and the loss of valuable organic matter. However, the potential for sustainable management of agricultural waste, particularly through composting, has gained significant attention in recent years. Composting offers a promising solution for converting waste into valuable resources, such as organic fertilizers, which can improve soil fertility and contribute to sustainable agroecosystems [1,2].

Corn cobs, a major by-product of corn production, represent a significant source of organic matter. These residues are primarily composed of cellulose, hemicellulose, and lignin, which contribute to their high carbon content but also make them resistant to decomposition [3]. Due to their lignocellulosic structure, corn cobs often pose challenges as substrates for composting, inhibiting microbial breakdown and reducing the overall efficiency of composting processes compared to other organic materials. Therefore, it is crucial to develop strategies that enhance the degradation of corn cobs and improve the quality of the resulting compost [4].

One such strategy is the reduction in particle size. The physical properties of organic matter, particularly particle size, significantly influence the rate of decomposition during composting. Smaller particles provide a larger surface area for microbial colonization, thereby enhancing microbial activity and facilitating the breakdown of complex organic compounds [5,6]. Previous studies have shown that reducing the particle size of lignocellulosic waste accelerates the composting process, enhances microbial access to the substrate, and improves the quality of the final compost [7]. However, the optimal particle size for composting corn cobs, one that balances decomposition efficiency with compost quality, remains unclear.

In addition to particle size reduction, the use of microbial inoculants has become a widely adopted practice to enhance the composting process. Microbial inoculants, typically composed of specific bacterial or fungal strains, serve as “starter cultures” that accelerate the breakdown of organic matter. These inoculants are known to enhance the thermophilic phase of composting, improve microbial diversity, and promote the rapid degradation of lignocellulosic materials [8,9]. Furthermore, microbial inoculants have the potential to improve the nutrient composition of the final compost product, enhancing its effectiveness as a fertilizer for agricultural use [10].

Despite these advantages, there is limited research on the impact of microbial inoculants specifically in the context of corn cob composting, and the interaction between microbial treatments and varying particle sizes has yet to be thoroughly explored. This study aims to investigate the effects of particle size and microbial inoculants on the aerobic decomposition and fertilizer efficiency of corn cobs during composting. Specifically, we examine three particle sizes (6, 10, and 20 mesh) and the application of a combination of bacterial and fungal communities as microbial inoculants as a “starter culture” to promote decomposition. Key parameters, including temperature, moisture content, and microbial activity, will be monitored throughout the composting process to determine the optimal combination of particle size and microbial treatment that maximizes both decomposition efficiency and the fertilizer value of compost.

The findings of this study will provide valuable insights into the role of particle size and microbial inoculants in optimizing corn cob composting, offering potential solutions for the sustainable management of agricultural waste. This research aligns with the principles of the circular economy, emphasizing the recycling of organic waste into valuable resources. It also supports global efforts to reduce reliance on synthetic fertilizers, which contribute to environmental pollution and impose significant economic burdens on farmers. By enhancing composting practices for corn cobs, this study aims to contribute to more sustainable agricultural systems and the effective utilization of agricultural waste [11].

## 2. Materials and Methods

### 2.1. Composting Materials and Experimental Design

In this study, corn cobs were collected as agricultural residues following the harvest of the Jin Qing 707 corn variety. The collected cobs were ground into three particle sizes, 6 mesh, 10 mesh, and 20 mesh, reflecting a range of sizes commonly used in composting and agricultural waste processing. Smaller particle sizes (6 mesh) increase the surface area, facilitating microbial colonization, while larger particle sizes (20 mesh) provide better aeration, preventing compaction and improving oxygen exchange. The intermediate size (10 mesh) offers a balance between surface area and porosity, promoting efficient decomposition and humification, which is in line with typical industry practices aimed at optimizing microbial activity and oxygen diffusion during the composting process.

To optimize the composting process, urea was added based on the organic matter content of the corn cobs (Organic Matter: 671.34 g/kg, Total Nitrogen: 3.54 g/kg) to adjust the carbon-to-nitrogen (C:N) ratio to 30:1. The moisture content was maintained between 65% and 70%, and microbial inoculants were applied to enhance the composting process. The inoculants used were Bacillus subtilis L-01 (14 g/kg) and Trichoderma harzianum L-02 (14 g/kg), with these dosages selected based on previous studies that demonstrated effective microbial activity and composting acceleration at these concentrations. The dosages were intended to enhance microbial diversity and support the decomposition of organic matter by introducing specialized microorganisms for lignocellulose degradation.

**Figure 1:**
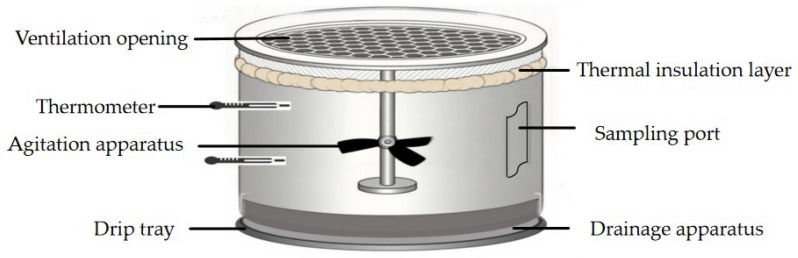
A schematic diagram of the composting apparatus.

The composting experiment was conducted in September 2024 at the experimental teaching base of Changchun University, located in Changchun, China. The corn cobs were divided into three groups based on particle size (M6, M10, and M20) for composting. Composting was carried out in specially designed reactors (Figure 1). This reactor employs natural ventilation. Each reactor contains approximately 3571 g of material, with three replicates per experimental group. The turning frequency is once every three days in the first 15 days and once every seven days after 15 days. Ventilation holes were placed at 5 cm intervals on the cover of each reactor, and drainage holes were installed at the diagonal corners of the reactor base. A ventilation pipe was centrally positioned to ensure proper airflow and drainage. Temperature monitoring probes were installed at three different positions in the reactor (top, middle, and bottom) to track temperature variations throughout the composting process. The compost heap was periodically turned as required.

**Figure 2:**
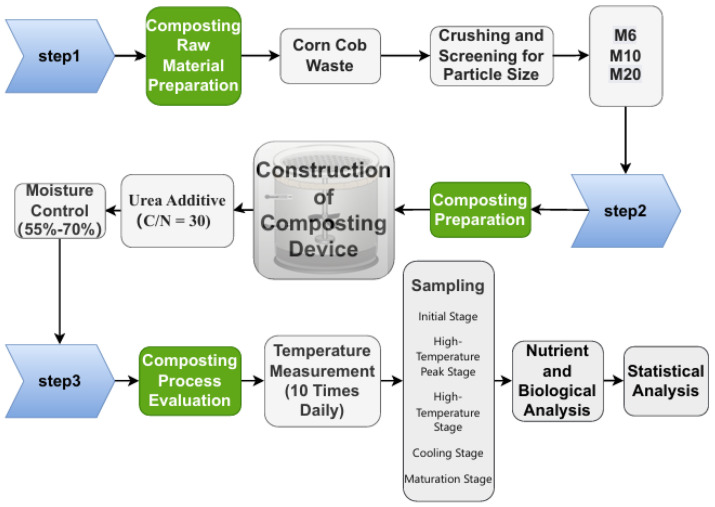
A Flowchart of the composting experiment treatments. (The arrows in the diagram serve to indicate the sequence of operational procedures.)

The composting process consisted of five distinct stages, the initial phase, thermophilic peak phase, thermophilic phase, cooling phase, and maturation phase, spanning a total duration of 51 days. Systematic sampling was conducted at each stage, with samples collected from the top, middle, and bottom portions of the compost heap and subsequently homogenized. The samples were then divided into two portions: one was air-dried and ground through a 0.25 mm sieve for the analysis of physical and chemical properties, humic substances (humic substance carbon [HSC], humic acid carbon [HAC], fulvic acid carbon [FAC]), and infrared spectroscopy analysis. The other portion was stored at −80 °C for microbiological analysis, with sampling times outlined in Table 1. The experimental procedure is illustrated in Figure 2.

### 2.2. Indicator Measurements

#### 2.2.1. Physicochemical Indicators

The pH was measured using a desktop pH meter (PHS–3C, Shanghai INESA Scientific Instrument Co., Ltd., Shanghai, China). Electrical conductivity was measured using a conductivity meter (DDS–307, Shanghai INESA Scientific Instrument Co., Ltd., Shanghai, China) [12]. Organic carbon content was determined by the potassium dichromate external heating method [13]. Total nitrogen was analyzed using a Kjeldahl nitrogen analyzer (K9840, Kjeltec, Foss Analytical, Hillerød, Denmark) [14], while total phosphorus was determined using a spectrophotometric method with an enzyme-linked immunosorbent assay (FLUOstar Omega, BMG LABTECH, Offenburg, Baden-Württemberg, Germany) [15]. Total potassium was measured using a flame photometric method (AA–6880, Shimadzu Corporation, Kyoto, Japan) [16]. Alkali-hydrolyzable nitrogen was determined using the diffusion method [17]. Available phosphorus was extracted with NaHCO_3_ and quantified by the molybdenum-antimony anti-colorimetric method. Available potassium was measured by ammonium acetate extraction followed by atomic absorption flame photometry [18]. Heavy metals were quantified using atomic absorption spectroscopy with a flame photometer (AA–6880, Shimadzu, Japan) [19]. Humic substances were extracted using an alkali extraction method [20], and their carbon content, as well as the components of humic substances, were measured using the potassium dichromate external heating method [21]. Humic acid (HA) was characterized by Fourier-transform infrared spectroscopy (FTIR) using an FTIR spectrometer (Is10, Thermo Fisher Scientific, Waltham, MA, USA) [22].

#### 2.2.2. Microbial Indicators

Microbial diversity was assessed using the Illumina NovaSeq sequencing platform with paired-end sequencing [23]. Bacterial sequences were primarily analyzed based on the 16S rRNA gene (V3 + V4 regions), while fungal sequences were analyzed based on the internal transcribed spacer (ITS) region, specifically ITS1_f [24]. Sequences and subsequent analysis were performed by Beijing Biomarker Technologies Co., Ltd., Beijing, China. For each microbial analysis, three biological replicates were performed. Each replicate was an independently sampled and processed sample to ensure the reliability and reproducibility of the results. The microbial diversity obtained in this study is closely related to compost maturity and nutrient availability. Higher microbial diversity during the composting process indicates a more complex and stable microbial community. Such a community can effectively decompose organic matter, accelerate the transformation of nutrients, and thus contribute to the maturity of compost.

### 2.3. Data Analysis

The data were statistically analyzed and organized using Microsoft Excel 365, with tables constructed accordingly. Statistical analysis was performed using SPSS 27.0, where one-way ANOVA and Duncan’s multiple range test were applied to analyze significant differences. Graphs were generated using OriginPro 2021, and partial least squares path modeling (PLS-PM) was conducted using R 4.3.0.

## 3. Results and Discussion

### 3.1. Basic Physicochemical Indicators During the Composting Process

#### 3.1.1. Temperature

**Figure 3:**
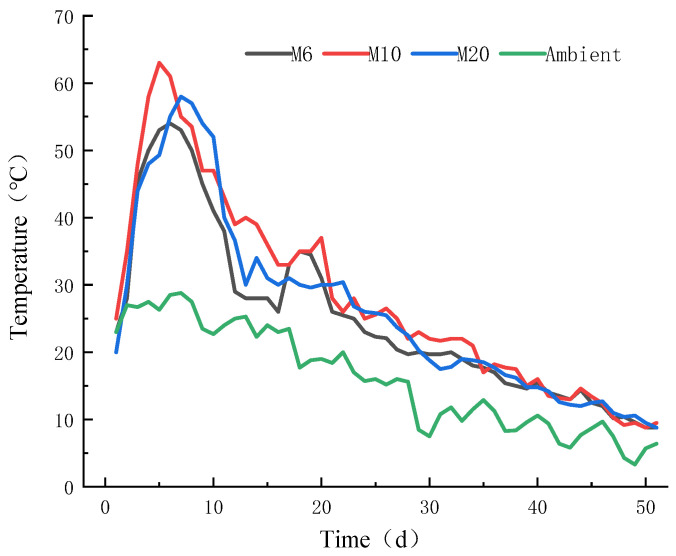
Variations in ambient temperature and pile temperature during the composting process.

Temperature variations during the composting process are important indicators of composting progress and quality. Figure 3 shows the temperature changes for each treatment throughout the composting process. Based on these, the composting process can be divided into four stages: the initial stage, thermophilic phase, cooling phase, and maturation phase. The M6, M10, and M20 treatments entered the thermophilic phase on the 4th, 4th, and 6th days, with maximum temperatures reaching 54 °C, 63 °C, and 58 °C. For all treatments, the temperature remained above 50 °C for five consecutive days, meeting the thermal requirements for compost maturity.

Among the three treatments, M6 and M10 entered the thermophilic phase the quickest, with M10 reaching the highest temperature. These differences likely stemmed from substrate structural traits and dynamic microbial shifts during stabilization. For example, particle size might have influenced aeration, thereby affecting temperature changes; dynamic microbial reorganization could involve shifts in dominant microbial populations at different stages [25]. The thermophilic phase in composting demonstrates a biological stimulation effect, where microorganisms intensify the mineralization of labile organic matter. The heat released during this process triggers a self-sustaining thermal feedback mechanism through exothermic metabolic cascades [26].

#### 3.1.2. pH Value, EC Value, and Organic Matter

**Figure 4:**
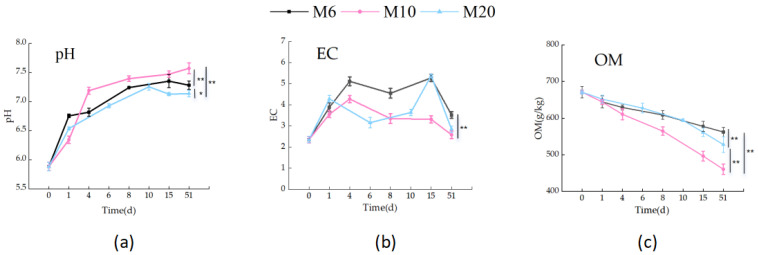
Changes in pH Value, EC Value, and Organic Matter (OM) in the three composting treatments: (**a**) changes in pH values during the composting process for the three treatments; (**b**) changes in EC values during the composting process for the three treatments; (**c**) Changes in organic matter during composting of the three treatments; * means *p* < 0.05, ** means *p* < 0.01. (The statistical significance assessment was conducted only for different treatment groups at the 51 day time point.).

pH is a critical factor in composting, governing microbial metabolism, organic matter mineralization, and nutrient retention. In this study, pH changes varied significantly with particle size (Figure 4a). The M10 treatment showed a more pronounced increase than M6 and M20, rising from an initial 5.9 to 7.6 after 51 days. This notable increase suggests that smaller particle sizes (such as 6 mm) promote more active microbial activity, leading to an enhanced release of alkaline substances, thereby accelerating the increase in pH during composting. The pH values of all treatments transitioned from neutral to alkaline during the composting process, primarily due to the microbial-mediated deacidification during organic matter mineralization [27]. intense microbial activity in the thermophilic phase further promoted this pH shift. Specifically, microbes like ammonia-oxidizing bacteria convert organic nitrogen to ammonium, which is then oxidized to nitrate. This process consumes hydrogen ions and depletes acidic substances, raising the pH [28].

Electrical conductivity (EC) indicates salt and ion concentrations in compost, crucial for assessing the process. As shown in Figure 4b, EC was monitored across three particle-size treatments. The initial EC of the corn cobs was 2.4 mS/cm, and all treatments remained below 4 mS/cm as composting progressed, indicating maturation in line with standards. EC variation correlated clearly with temperature: it rose during heating and fell during cooling. In the thermophilic phase, intense microbial activity decomposes organic matter, converting it into mineral salts. Their dissolution increased ion concentration, raising EC [29]. Conversely, during the cooling phase, decreased microbial activity altered nitrogen metabolism. This changed ionic composition, as cations bound with nitrate ions, reducing EC [30].

The organic matter (OM) content is a critical indicator of composting efficiency. As shown in Figure 4c, the OM content of three particle size treatments (M6, M10, M20) was monitored over 51 days. Starting near 700 kg, OM decreased differentially across treatments. M6 (6 mm) declined steadily to 650 kg, suggesting smaller particles improve microbial colonization and decomposition. M10 (10 mm) showed the most pronounced reduction, to 500 kg, highlighting the efficiency of medium sized particles. Statistical analysis confirmed that M10 had the most significant OM loss (*p* < 0.05). In contrast, M20 (20 mm) decreased the least, to only 550 kg, indicating that larger particles limit microbial access and slow decomposition [31]. These results confirm that smaller particles enhance composting efficiency, while larger ones impede it, aligning with previous studies [32,33].

During the composting process, pH value, electrical conductivity (EC), and organic matter (OM) content are all important indicators. The pH value changes with particle size, shifting from acidic to alkaline, which reflects the fact that the compost is approaching maturity and is more conducive to the activity of beneficial microorganisms. EC is related to temperature, rising first and then decreasing, and its change reflects the stability and maturity of the compost. The OM content decreases during composting. The smaller the particle size, the higher the decomposition efficiency, and the degree of OM reduction reflects the maturity of the compost. These indicators are interrelated and jointly outline the overall picture of the composting process and maturity, which is of great significance for guiding fertilization.

#### 3.1.3. Nutrient Content During the Composting Process

**Figure 5:**
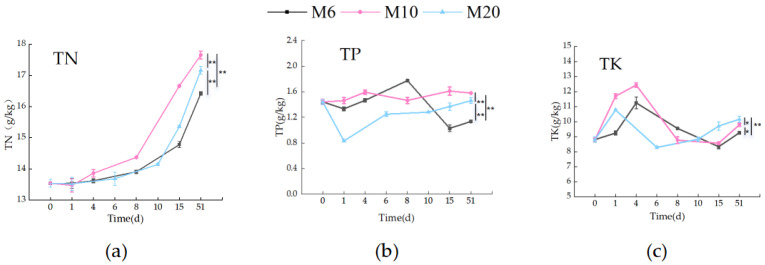
Changes in total nitrogen, phosphorus, and potassium in the three treatments: (**a**) changes in total nitrogen during the composting process for the three treatments; (**b**) changes in total phosphorus during the composting process for the three treatments; (**c**) changes in total potassium during the composting process for the three treatments; * means *p* < 0.05, ** means *p* < 0.01. (The statistical significance assessment was conducted only for different treatment groups at the 51 day time point.).

Total nitrogen (TN) is crucial for maintaining soil nutrient balance and promoting plant growth in aerobic composting. As composting progressed, TN content gradually accumulated (Figure 5a). The M10 treatment showed the highest increase, with TN rising by 4.11 g/kg to 17.65 g/kg, while M20 and M6 showed smaller increases. The C/N ratios at day 51 were 19.87 for M6, 17.85 for M10, and 15.13 for M20, indicating that smaller particle sizes favor TN accumulation. Smaller particles provide a larger surface area for nitrogen-transforming microorganisms, enhancing nitrogen fixation and accelerating the decomposition of nitrogen-rich organic matter [34]. Additionally, smaller particles improve contact with moisture and oxygen, facilitating microbial activity and nitrogen cycling. The lower C/N ratio in smaller particles suggests more efficient microbial utilization of carbon sources, resulting in better nitrogen retention and accumulation [35].

Total Phosphorus (TP) is essential for compost quality and soil fertility. Throughout the composting process, TP content fluctuated by approximately 0.5 g/kg, with no significant changes, indicating high phosphorus stability. At the end of composting, M6 had the lowest TP content at 1.13 g/kg. Larger particle sizes, such as M20, provide a looser structure that enhances oxygen diffusion and microbial activity, leading to phosphorus transformation. M10 showed balanced microbial activity, while M20’s larger particles limited oxygen diffusion, maintaining relatively stable TP content (Figure 5b). In contrast, the M10 and M20 treatments showed TP contents that remained close to their initial values by the end of the composting process. This may be due to the balanced microbial activity and phosphorus transformation in M10, where particle size was optimized. On the other hand, the larger particle size in M20 limited oxygen diffusion, thereby reducing excessive microbial phosphorus utilization and maintaining a relatively stable phosphorus content. Despite the varying changes in TP content across different particle sizes, overall data from the composting process indicated that particle size had no significant impact on TP content. This could be due to the complex interplay of various factors during composting, which may have masked the isolated effect of particle size on TP content. For example, microbial metabolic pathways could change in response to different particle sizes, affecting the direction and extent of phosphorus transformation. Furthermore, slight fluctuations in environmental temperature could alter the phosphorus speciation, and changes in humidity could influence phosphorus dissolution and migration within the compost system. These interacting factors likely caused the changes in TP content to show no clear correlation with particle size [36,37].

Total Potassium (TK) is relatively stable in composting due to its low volatility. The data showed an increase in TK content during the thermophilic phase, with a peak followed by a decrease as composting progressed (Figure 5c). At the end of the process, the M20 treatment had the highest TK content (10.16 g/kg), while M6 and M10 had 9.26 g/kg and 9.79 g/kg, respectively. This indicates that potassium remained stable throughout the composting process, crucial for maintaining soil fertility. Stable TK content ensures that compost can provide a consistent potassium supply, supporting plant growth and biochemical processes such as enzyme activation and osmotic pressure regulation [38,39].

**Figure 6:**
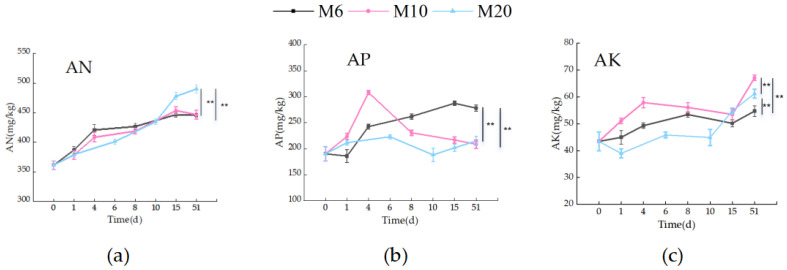
Changes in available nitrogen, available phosphorus, available potassium the three treatments: (**a**) changes in available nitrogen during the composting process for the three treatments; (**b**) changes in available phosphorus during the composting process for the three treatments; (**c**) changes in available potassium during the composting process for the three treatments; ** means *p* < 0.01. (The statistical significance assessment was conducted only for different treatment groups at the 51 day time point.).

As shown in Figure 6a, the Available Nitrogen (AN) content during the corn cob aerobic composting process increased gradually across all treatments, with particle size significantly affecting the accumulation. At the start, AN content was approximately 350 mg/kg for all treatments. Over time, AN content rose steadily, with the M10 treatment showing the most significant increase, reaching approximately 450 mg/kg by day 51. In comparison, M6 and M20 treatments saw more moderate increases, with final values of 420 mg/kg and 430 mg/kg, respectively. The higher increase in AN in the M10 treatment can be attributed to the optimal particle size, which enhanced microbial activity and facilitated more efficient nitrogen mineralization and nitrification. Smaller particles provide a greater surface area for microbial colonization, thereby accelerating nitrogen cycling and improving nitrogen availability. While the M6 treatment also saw an increase in AN, the rise was slower, suggesting that smaller particle sizes might limit certain microbial processes despite enhancing others. On the other hand, the M20 treatment, with the largest particle size, exhibited the least increase in AN content, likely due to reduced microbial access to the organic material, which hindered nitrogen transformation [40]. These results are consistent with previous studies that highlight the importance of particle size in optimizing nitrogen release during composting, where smaller particles generally promote better microbial breakdown of organic matter and enhance nitrogen cycling, leading to higher AN content in the compost [41,42].

As shown in Figure 6b, the Available phosphorus (AP) content increased progressively during the composting process across all three particle size treatments. The M10 treatment exhibited the most significant increase in AP content, rising from approximately 40 mg/kg to nearly 70 mg/kg by the end of the 51-day composting period. In comparison, the M6 and M20 treatments showed smaller increases, reaching final AP contents of 55 mg/kg and 50 mg/kg, respectively. The statistical comparison of the treatments, presented in Figure 6b, indicates that M10 resulted in the highest increase in AP content, significantly higher than both M6 and M20 (*p* < 0.05). The more pronounced increase in AP content observed in the M10 treatment can be attributed to the optimized particle size, which enhanced microbial activity and phosphorus mineralization. Smaller particle sizes provide a larger surface area, promoting better microbial colonization and increasing the efficiency of phosphorus cycling [43]. The M6 treatment, despite having a smaller particle size, did not show as significant an increase in AP content, suggesting that factors such as microbial community structure and environmental conditions also play a role in phosphorus transformation. The M20 treatment, with the largest particle size, showed the least increase in AP content, likely due to limited microbial access and reduced aeration, which hindered the phosphorus transformation process [44].

As shown in Figure 6c, the Available Potassium (AK) content increased gradually during the composting process across all three treatments. At the beginning, the AK content was around 200 mg/kg for all treatments. Over the composting period, the AK content in the M6 treatment showed the most significant increase, reaching nearly 300 mg/kg by day 51, while the M10 and M20 treatments exhibited more moderate increases, with final AK contents of approximately 270 mg/kg and 240 mg/kg, respectively. The statistical comparison in Figure 6c reveals significant differences between the treatments, with M6 showing the highest increase in AK content (*p* < 0.05), followed by M10 and M20, which showed similar but lower increases. The rapid increase in AK content in the M6 treatment can be attributed to the smaller particle size, which enhanced microbial activity and facilitated the release and cycling of potassium from organic matter. The M6 treatment likely benefited from better microbial colonization and greater surface area for microbial interactions. In contrast, M10 and M20, with larger particle sizes, showed less significant increases in AK content. The slower increase in AK content in these treatments may be due to limited microbial access to organic substrates in the larger particles, leading to slower potassium release [45]. These findings highlight the importance of particle size in the release of available potassium during composting. Smaller particle sizes, such as in the M6 treatment, enhance microbial breakdown and the bioavailability of nutrients like potassium, whereas larger particle sizes (M10 and M20) reduce microbial accessibility, slowing down nutrient cycling [46].

### 3.2. Changes in Humic Substances Components During Composting

**Figure 7:**
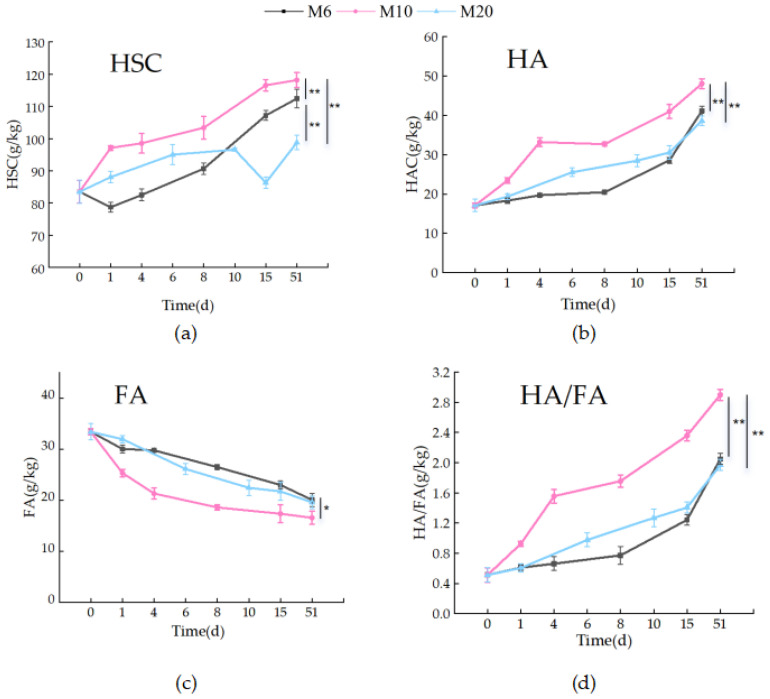
Carbon changes in humic substances for the three treatments: (**a**–**d**) Variation trends of HSC, HAC, FAC and HAC/FAC, during the three composting processes; * means *p* < 0.05, ** means *p* < 0.01 (The statistical significance assessment was conducted only for different treatment groups at the 51-day time point.)

Transformations in humic substance carbon (HSC), particularly humic acid carbon (HAC) and fulvic acid carbon (FAC), are key indicators of compost stabilization and maturity. Across all treatments, HAC increased while FAC declined (Figure 7a–d), reflecting the progressive conversion of labile compounds into stable humic structures.

#### 3.2.1. Humic Substance Carbon Transformations (HSC, HAC, FAC)

Among treatments, M10 showed the most efficient humification, with HAC rising from 23.44 g/kg to 48.30 g/kg, followed by M6 (41.61 g/kg) and M20 (38.16 g/kg). Simultaneously, FAC decreased most rapidly in M10, resulting in the highest HAC/FAC ratio. This indicates enhanced microbial-mediated oxidative polymerization and humic acid formation [47]. Previous studies have also reported similar trends in humic substance decomposition during organic matter composting. A similar increase in HAC and decrease in FAC were observed, indicating a common pattern in the transformation of humic substances during decomposition.

The superior humification in M10 can be attributed to its balanced structural properties. The 10-mesh size likely provided sufficient surface area for microbial colonization while maintaining pore connectivity for oxygen diffusion, conditions essential for aerobic humification pathways [48]. In contrast, finer particles (M6) may have promoted compaction and localized anaerobic conditions, while coarser particles (M20) reduced microbial accessibility and slowed organic matter decomposition.

These findings highlight particle size as a decisive physical regulator of carbon stabilization during composting. The optimal humification performance of M10 underscores the synergy between substrate structure and microbial metabolism, emphasizing that appropriate particle refinement is necessary to maximize humic acid accumulation and compost value [49].

The transformation of humic substance carbon (HSC) components, specifically humic acid carbon (HAC) and fulvic acid carbon (FAC), is a crucial process determining the stability and agronomic value of the final compost. Our results revealed that the particle size of corncob significantly influenced the dynamics of these carbon pools.

Throughout the composting process, a remarkable increase in HAC and a concurrent decrease in FAC were observed across all treatments. This trend indicates the continuous humification and maturation of organic matter. Specifically, the HAC content in the M10 treatment increased from an initial 23.44 g/kg to a final 48.30 g/kg, achieving the highest net gain and the final value among all treatments. The M6 treatment also exhibited a substantial increase in HAC, reaching 41.61 g/kg, while the M20 treatment yielded the lowest final HAC content of 38.16 g/kg.

Correspondingly, the FAC content showed a consistent decline. The initial FAC values were similar across particle sizes, but the degradation rate and final extent varied. The M10 treatment facilitated the most efficient reduction of FAC, followed by M6 and M20. The ratio of HAC to FAC, and the progressive increase in the HSC values, further confirm the advancement of humification. The M10 treatment consistently demonstrated the most pronounced transformation, ending with the highest HSC and the most favorable HAC/FAC ratio.

The systematic increase in HAC at the expense of FAC is a hallmark of successful composting. This transformation signifies the microbial-driven conversion of simpler, bioavailable fulvic acids into more complex, stable, and recalcitrant humic acids, which are central to long-term carbon sequestration in soil [47]. The observed dynamics underscore that composting effectively stabilizes organic carbon from the labile FAC pool into the stable HAC pool. The superior performance of the M10 treatment in accumulating HAC can be attributed to the creation of an optimal microenvironment for humification. The 10-mesh particle size likely established an ideal balance between substrate surface area for microbial colonization and inter-particle porosity for oxygen diffusion. Sufficient oxygen is critical for the aerobic microorganisms that catalyze the oxidative polymerization reactions required for HAC synthesis [48]. While finer particles (M6) offer greater surface area, they risk compacting and creating anaerobic pockets that hinder these aerobic processes. Conversely, larger particles (M20) limit microbial access and reduce the overall decomposition efficiency, thereby retarding the humification pathway.

The added microbial inoculant presumably acted synergistically with this optimal physical structure in M10. Enhanced aeration would have sustained robust metabolic activity of both the inoculated and indigenous microbial communities, driving the continuous conversion of FAC and other intermediate products into HAC. This synergy between physical pretreatment (size reduction) and biological intervention (inoculation) is a key finding of this study, demonstrating that the efficacy of microbial additives is contingent upon the physical properties of the feedstock [49]. The lower HAC yield in the M20 treatment highlights a physical constraint that the microbial consortium could not overcome, emphasizing the paramount importance of feedstock preparation in composting science and technology. In conclusion, the particle size of corncob is a decisive factor in steering the carbon flow during composting towards the formation of stable humic acids. The identification of the 10-mesh size as the optimal particle size in this study highlights the significance of optimizing particle size (around 10-mesh) for improving the performance of future industrial decomposition systems. This finding can guide the design and operation of industrial composting facilities, aiming to enhance the efficiency of humic acid formation and carbon sequestration, and ultimately produce high-quality compost products for various agricultural and environmental applications.

#### 3.2.2. Evolution of Humification Degree and Compost Maturity (HA/FA)

The humification degree, assessed by the HA/FA ratio, increased steadily in all treatments, confirming effective compost maturation (Figure 7). Initial HA/FA values (<1.0) reflected fresh organic substrates, whereas final values exceeded the maturity threshold of 1.5–1.7 [50].

M10 again demonstrated the greatest enhancement, with HA/FA increasing from ~0.91 to 2.85, outperforming M6 (2.06) and M20 (1.96). This superior humification kinetic suggests that the 10-mesh structure provided optimal aeration and microbial access, facilitating oxidative polymerization of FAC into HAC [51]. Restricted aeration in M6 and limited biodegradability in M20 likely constrained their humification efficiency [52].

Collectively, these results confirm that corn cob particle size substantially influences humification pathways and compost maturity. The 10-mesh particle size most effectively promoted stable humus formation, producing compost with enhanced agricultural value and greater carbon sequestration potential.

#### 3.2.3. FTIR Spectroscopic Analysis of Humic Acids During Composting

**Figure 8:**
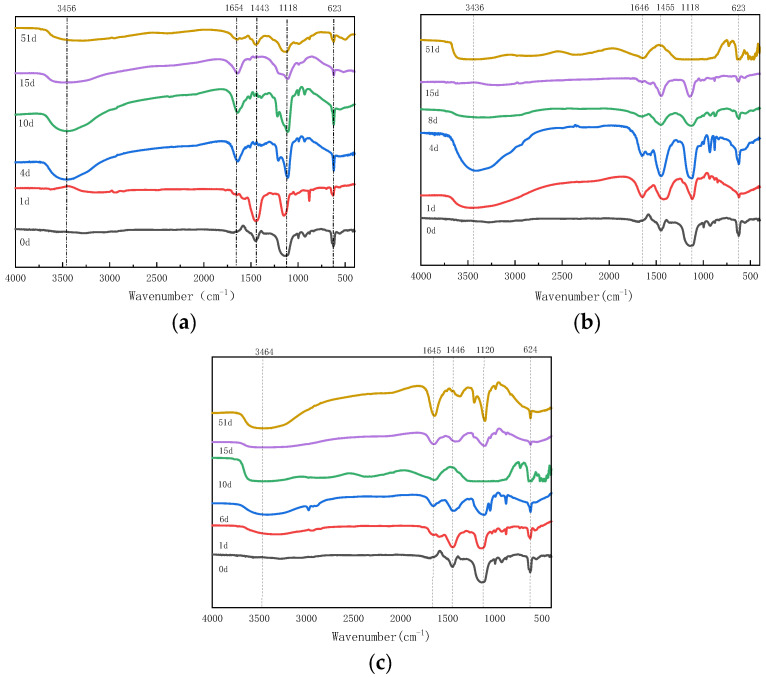
FTIR analysis of humic acids (HA) during composting under three treatments: (**a**) HA from the M6 treatment. (**b**) HA from the M10 treatment. (**c**) HA from the M20 treatment.

The FTIR spectra of HA from all treatments (M6, M10, M20) exhibited characteristic absorption bands, confirming the heterogeneous nature of humic substances (Figure 8). The key bands observed were: a broad band around 3440–3450 cm^−1^ attributed to O-H stretching; a band at 1645–1650 cm^−1^ related to C=O stretching of amide groups (amide I) or aromatic C=C; a peak near 1450 cm^−1^ assigned to aliphatic C-H deformations; a band at 1120 cm^−1^ indicative of C-O stretching of polysaccharides or ethers; and a band at 623–624 cm^−1^ corresponding to out-of-plane bending of aromatic C-H. As composting progressed, the spectra showed an increase in aromatic structures, particularly in the M10 treatment, with the intensity of the aromatic C-H bending band (~623 cm^−1^) relative to the aliphatic C-H band (~1450 cm^−1^) becoming more pronounced, suggesting efficient aromatic enrichment.

These changes align with the classical humification pathway, where labile, aliphatic, and polysaccharide-like compounds are degraded, while recalcitrant, aromatic structures are preserved and synthesized. Notably, the increase in the aromatic/aliphatic ratio further supports the advancement of compost maturity [53]. The more prominent aromatic condensation observed in the M10 treatment indicates superior humification, likely due to the optimal oxidative conditions provided by the 10-mesh particle size, which enhanced oxygen supply and microbial activity. This, in turn, facilitated more efficient aromatic polymerization, leading to higher aromatic content. In contrast, the M6 and M20 treatments showed less efficient aromatic condensation, as indicated by their respective FTIR spectra, likely due to oxygen limitations in M6 and slower decomposition in M20.

Recent studies on FTIR decomposition during fermentation have highlighted similar trends, further supporting these findings [54]. This affirms the role of particle size and microbial activity in driving the humification process.

### 3.3. Assessment of Compost Quality: Phytotoxicity and Heavy Metal Safety

The agronomic value and environmental safety of the final compost products were evaluated by the seed germination index (GI) and the concentrations of key heavy metals. These parameters are critical determinants for the practical application of compost in agriculture (Table 2).

As presented in Table 2, the germination index (GI) varied significantly among the three treatments. The M10 compost yielded the highest GI of 93.63%, which is well above the widely accepted maturity threshold of 80% [55]. The GI values for M6 and M20 composts were 91.52% and 88.54%, respectively, also indicating acceptable levels of maturity but being significantly lower than that of M10. A GI above 90% signifies the effective degradation of phytotoxic substances, such as ammonia, volatile organic acids, and phenolic compounds, which are typically present in the initial phases of composting [56]. The optimal composting conditions in the M10 treatment, characterized by efficient oxygenation, likely facilitated the rapid microbial degradation of these phytotoxins, thereby resulting in a compost that is non-inhibitory and even beneficial to plant growth.

Concurrently, the heavy metal analysis revealed a clear and consistent pattern across all five elements monitored (Pb, Cd, Cr, As, Hg). The M10 compost consistently exhibited the lowest concentrations of heavy metals. For instance, the Pb content in M10 (9.4 mg/kg) was substantially lower than in M6 (16.2 mg/kg) and M20 (21.3 mg/kg). Similarly, the Cd content in M10 (0.6 mg/kg) was only about one-third of that in M20 (2.5 mg/kg). All measured heavy metal concentrations across the treatments were below the maximum limits stipulated by the Chinese organic fertilizer standard (NY/T 525-2021), indicating overall safety [57]. A vigorous and prolonged thermophilic phase, which was presumably best achieved in the well-aerated M10 pile, can lead to greater microbial biomass production and the subsequent formation of stable humic substances with high cation exchange capacity (CEC) and numerous functional groups [58]. These humic components effectively immobilize and complex heavy metal ions, reducing their bioavailability and potential leachability. In contrast, the less efficient humification in the M20 treatment may have resulted in a lower capacity for metal complexation. Furthermore, the intense microbial activity in M10 could have contributed to the volatilization of certain metals like Hg [59].

### 3.4. Dynamics of Bacterial Community Structure and Diversity During Composting

High-throughput sequencing of the bacterial 16S rRNA gene was performed to investigate the microbial mechanisms driving the differential composting outcomes influenced by corn cob particle size. Samples were collected from the initial (MA1, MB1, MC1), thermophilic (MA2, MB2, MC2), and curing (MA3, MB3, MC3) phases, corresponding to the M6, M10, and M20 treatments, respectively.

Alpha-diversity indices (ACE, Chao1, Simpson, and Shannon) showed a steady increase in bacterial richness and diversity from the initial to curing phase across all treatments, with the extent of this increase being size-dependent (Table 3). The M10 treatment developed the most diverse and stable bacterial community, reaching the highest Shannon index (7.61) and richness (ACE: 926.76) at the curing phase, while M6 and M20 showed slower microbial succession, reflecting suboptimal aeration and delayed colonization.

At the phylum level, Firmicutes dominated the early and thermophilic phases due to their thermotolerance and cellulolytic activity [60]. During the curing phase, Actinobacteriota and Proteobacteria became predominant, especially in the M10 treatment, contributing to the degradation of lignocellulosic residues and humic substance formation [61]. At the genus level, the relative abundance of Thermobifida and Streptomyces increased markedly, confirming their key roles in oxidative decomposition and humification [62].

**Figure 9:**
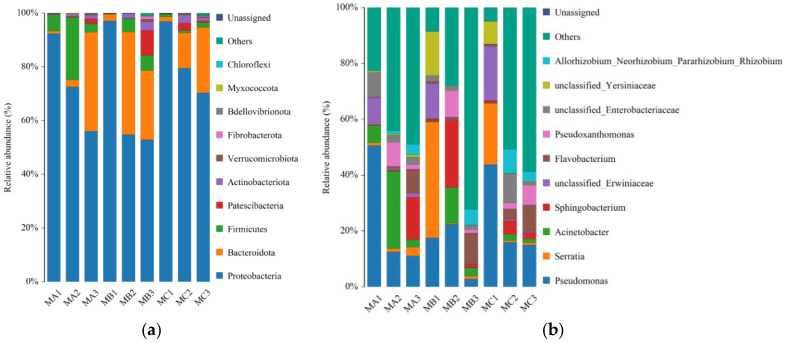
Corncob particle size drives bacterial community succession during composting. (**a**) phylum-level and (**b**) genus-level bacterial community composition across different composting phases for M6, M10, and M20 treatments.

These community shifts are tightly coupled with the physicochemical microenvironment shaped by particle size. The 10-mesh fraction provided an optimal balance between surface area and porosity, promoting oxygen diffusion and sustaining aerobic metabolism. This environment favored aerobic decomposers and humus-forming bacteria while limiting anaerobic taxa. Genera such as Streptomyces, known for secreting lignin-degrading enzymes (laccase, peroxidase), were especially enriched in M10, supporting enhanced humification efficiency [63]. In contrast, the finer M6 particles likely caused compaction and restricted aeration, whereas the course M20 fraction delayed microbial colonization and organic matter decomposition (Figure 9).

### 3.5. Dynamics of Fungal Community Structure and Diversity During Composting

Alpha-diversity indices revealed distinct particle size-dependent patterns in fungal community development (Table 4). While all treatments exhibited increasing fungal richness (ACE and Chao1 indices) and diversity (Shannon index) throughout the composting process, the M10 treatment demonstrated the most pronounced enhancement, achieving significantly higher values in the curing phase (ACE: 926.76; Chao1: 926.75; Shannon: 7.61) compared to other treatments. This superior diversity progression aligns with the optimal physical structure created by the 10-mesh particles, which apparently supported more robust fungal colonization and succession.

**Figure 10:**
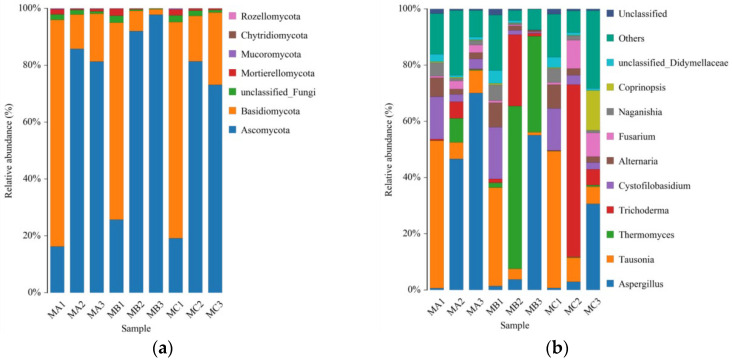
Corncob particle size drives fungal community succession during composting. (**a**) phylum-level and (**b**) genus-level fungal community composition across different composting phases for M6, M10, and M20 treatments.

The fungal community composition shifted significantly across phases (Figure 10). Ascomycota dominated the initial stages, reflecting their role in primary organic matter breakdown [64]. Notably, the M10 treatment showed the most dramatic community transition during the curing phase, with a marked increase in Basidiomycota, known for its lignocellulose-degrading capacity and involvement in humification processes [65].

At the genus level, the M10 treatment notably enriched taxa with lignocellulose-degrading abilities, including Aspergillus and Trichoderma species, which are key to efficient humic substance formation [66]. This strategic enrichment in functional genera underscores the critical role of particle size in promoting microbial functional diversity and improving compost maturity, directly linking fungal selection to composting efficiency.

### 3.6. Correlation Between Microbial Community Structure and Nutrient Indicators

**Figure 11:**
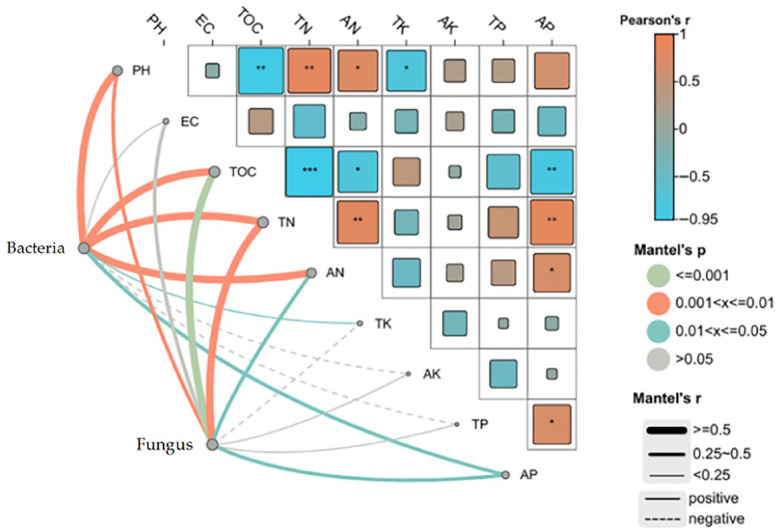
The Correlation between Microbial Community Structure and Composting Indicators. Note: This figure illustrates the Pearson correlation coefficients (r) and Mantel’s *p* values for the relationships between microbial community structures (Bacteria and Fungi) and various composting indicators, including pH, EC, total organic carbon (TOC), total nitrogen (TN), ammonium nitrogen (AN), total potassium (TK), available potassium (AK), available phosphorus (AP), and total phosphorus (TP) during the composting process. The size and color of the squares indicate the strength and direction of the correlation (positive or negative), with statistical significance marked by different Mantel’s *p* value ranges. * Means *p* < 0.05, ** means *p* < 0.01, and *** means *p* < 0.001. These results highlight the intricate interactions between microbial dynamics and nutrient cycling, which are influenced by particle size and composting conditions.

Understanding the relationships between microbial community dynamics and compost quality is crucial for optimizing the composting process and improving the final product. The heatmap (Figure 11) illustrates the correlations between key microbial community structures (Bacteria and Fungus) and various composting indicators, such as pH, electrical conductivity (EC), total organic carbon (TOC), total nitrogen (TN), ammonium nitrogen (AN), total potassium (TK), available potassium (AK), available phosphorus (AP), and total phosphorus (TP). From the heatmap, it is evident that the microbial community, particularly bacteria, showed significant correlations with multiple composting indicators. The positive correlations between bacterial abundance and TOC, TN, and AN (Pearson’s r ≥ 0.5) suggest that bacteria are actively involved in the decomposition of organic matter and nitrogen transformation, contributing to the cycling of key nutrients during composting. Notably, the strong positive correlation between bacteria and AN, as well as the moderate correlation with TN, supports the critical role of bacteria in ammonium mineralization and nitrogen cycling, processes essential for maintaining nutrient availability in the compost [67].

On the other hand, fungi exhibited weaker correlations with most of the composting indicators, though a significant negative correlation was observed between fungal abundance and available phosphorus (AP) (Pearson’s r < −0.5). This suggests that fungi may be involved in phosphorus mineralization or immobilization processes, which could influence the bioavailability of phosphorus during composting [68]. Interestingly, the correlation patterns between microbes and nutrients varied with particle size, indicating that different particle sizes may influence microbial community composition and nutrient dynamics. The M10 treatment, with an optimal particle size, exhibited the strongest correlations between bacteria and nutrient cycling, suggesting that the microbial community in this treatment was most efficient in nutrient transformation [69]. These findings underline the complex interactions between microbial communities and composting conditions. They also highlight the importance of particle size in influencing microbial activity and nutrient cycling. The correlation patterns observed in this study emphasize the need to carefully manage particle size and microbial composition to optimize composting efficiency and improve the nutritional value of the compost, ultimately enhancing its effectiveness as a soil amendment [70].

### 3.7. Structural Equation Model of the Degree of Humification During Composting

**Figure 12:**
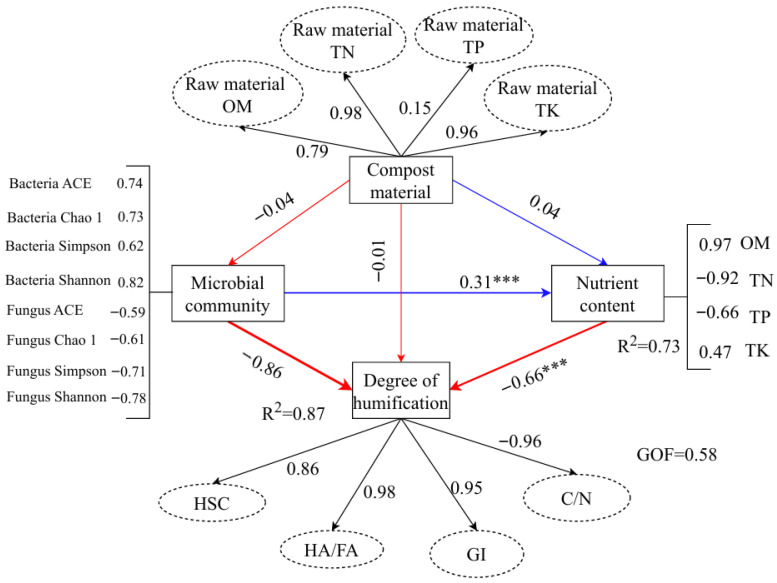
The Structural Equation Model of the Degree of Humification in the Composting Process. *** means *p* < 0.001. The red line indicates the positive path, and the blue line indicates the negative path. The width of the line indicates the degree of influence. The values next to the lines are the path coefficients, and the dashed lines indicate insignificant effects. R2 represents the proportion of the explained variance. Microbial community diversity was expressed using the α-diversity index. The degree of humification was indicated by humification indicators (HSC content, HA/FA, GI value, and C/N).

The structural equation model (SEM) in Figure 12 illustrates the complex relationships between compost material properties, microbial community dynamics, nutrient content, and the degree of humification during the composting process. The model shows that microbial community structure significantly impacts the degree of humification, with bacteria showing a stronger influence than fungi, as evidenced by the higher correlation coefficients for bacterial diversity indices (Bacteria ACE, Chao 1, Simpson, Shannon) compared to fungal indices (Fungus ACE, Chao 1, Simpson, Shannon) [71]. The negative relationship between the microbial community and the degree of humification (−0.86) indicates that increased microbial diversity, especially bacteria, contributes to the stabilization and transformation of organic matter into more stable humic substances [72].

Humification is closely tied to compost material properties, positively correlated with HSC, HA/FA, and GI (R^2^ = 0.87, 0.98, 0.95). This suggests that the extent of organic matter transformation into humic substances is governed by microbial activity, with more decomposed organic material leading to higher HSC and GI values [73,74]. Additionally, the structural model reveals the significant role of nutrient content in shaping composting outcomes. Nutrient content, particularly total organic matter (OM), total nitrogen (TN), and total potassium (TK), is positively correlated with the degree of humification, suggesting that a balanced nutrient profile promotes the formation of stable humic substances during composting [75].

Furthermore, the nutrient content was strongly influenced by the raw material composition, with total nitrogen (TN) and total phosphorus (TP) showing significant positive correlations with the microbial community and humification (R^2^ = 0.92 and 0.66, respectively). This emphasizes the importance of initial composting material properties in determining the efficiency of humification and the overall quality of the compost [76]. The model’s good-of-fit (GOF = 0.58) confirms the robustness of the relationships and the suitability of the SEM for understanding the dynamics of composting [77].

In conclusion, this structural equation model effectively highlights the complex interactions between microbial community structure, compost material, nutrient cycling, and humification, providing valuable insights into optimizing composting processes to achieve high-quality compost for soil fertility improvement [78].

## 4. Conclusions

This study offers insights into how particle size and microbial inoculants affect corn cob composting. Particle size impacts composting rate, microbial community, and humic substance formation. The M10 treatment excelled in humification, microbial activity, and nutrient cycling. Smaller M20 particles aid microbial colonization, but M10’s optimal size maximized microbial efficiency, increasing HSC and HAC. Larger M6 particles slowed microbial activity and humic substance transformation.

Microbial inoculants, particularly Bacillus subtilis and Trichoderma harzianum, enhanced the composting process by promoting microbial diversity and accelerating the decomposition of lignocellulosic materials. The structural equation model (SEM) highlighted the significant role of microbial community structure in influencing nutrient content and the degree of humification, showing strong correlations between bacterial diversity and key nutrients such as total nitrogen (TN) and ammonium nitrogen (AN).

However, it is important to acknowledge a limitation in experimental design. This study did not include a “no microbial inoculants” control group, which means we cannot fully isolate the impact of microbial inoculants on the composting process. All treatment groups included microbial inoculants, and thus, the observed effects may reflect a combination of particle size and microbial activity. Future studies should include a control group with no microbial inoculants to better understand the specific contributions of microbial communities to composting efficiency and humification.

In summary, optimizing particle size and inoculation can improve composting, humification, and nutrient retention. This study underscores their importance in enhancing compost quality, offering a sustainable alternative to synthetic fertilizers for soil health and fertility in agriculture.

## Figures and Tables

**Table 1:** Sampling times during the composting process.

Sample Collection	M6	M10	M20
Initial Stage	1d (9.24)	1d (9.24)	1d (9.24)
Peak Thermophilic Stage	4d (9.27)	4d (9.28)	6d (9.30)
Thermophilic Stage	8d (10.1)	8d (10.1)	10d (10.4)
Cooling Stage	15d (10.8)	15d (10.8)	15d (10.9)
Curing Stage	51d (11.13)	51d (11.13)	51d (11.13)

**Table 2:** The Seed germination index (GI) and concentrations of heavy metals in the final compost products from treatments M6, M10, and M20.

Samples	Germination Index (%)	Pb (mg/kg)	Cd (mg/kg)	Cr (mg/kg)	As (mg/kg)	Hg (mg/kg)
M6	91.52 b	16.2 ± 2.3 a	1.9 ± 0.4 a	41.7 ± 4.5 a	4.1 ± 0.6 a	0.7 ± 0.1 ab
M10	93.63 a	9.4 ± 1.1 b	0.6 ± 0.2 b	28.3 ± 3.8 b	1.9 ± 0.2 b	0.3 ± 0.08 b
M20	88.54 c	21.3 ± 3.0 c	2.5 ± 0.4 c	59.4 ± 5.5 c	5.8 ± 0.9 c	1.1 ± 0.2 a

Note: Different letters denote significant differences among the composting treatments (*p* < 0.05).

**Table 3:** The Statistical Alpha Diversity Indices of Bacteria in the Sample.

Samples	ACE Index	Chao1 Index	Simpson Index	Shannon Index
MA1	400.87 ± 8.35 Ba	400.37 ± 8.52 Ba	0.91 ± 0.07 Ba	5.10 ± 0.16 Ca
MA2	387.83 ± 10.35 Ba	388.33 ± 10.84 Ba	0.91 ± 0.05 Ba	5.71 ± 0.23 Bb
MA3	455.22 ± 7.65 Ab	455.36 ± 11.84 Ab	0.99 ± 0.03 Aa	7.86 ± 0.32 Aa
MB1	390.02 ± 10.77 Ca	392.17 ± 12.86 Ca	0.80 ± 0.05 Bb	3.87 ± 0.18 Cb
MB2	492.16 ± 13.7 Bc	493.75 ± 9.86 Bc	0.93 ± 0.06 Aa	5.53 ± 0.35 Bb
MB3	926.76 ± 12.52 Aa	926.75 ± 12.16 Ab	0.98 ± 0.06 A	7.61 ± 0.49 Aa
MC1	306.20 ± 11.52 Cb	307.0 ± 8.65 Cb	0.89 ± 0.05 Aa	4.20 ± 0.23 Cb
MC2	414.29 ± 11.32 Bb	414.5 ± 9.53 Ba	0.98 ± 0.07 Aa	7.00 ± 0.14 Ba
MC3	914.21 ± 12.52 Aa	914.66 ± 13.54 Aa	0.98 ± 0.08 Aa	7.88 ± 0.26 Aa

Note: Different uppercase letters indicate significant differences between composting phases within the same treatment, while different lowercase letters indicate significant differences between treatments at the same phase (*p* < 0.05).

**Table 4:** The Statistical Alpha Diversity Indices of fungal in the Sample.

Samples	ACE Index	Chao1 Index	Simpson Index	Shannon Index
MA1	400.87 ± 8.35 Ba	400.37 ± 8.52 Ba	0.91 ± 0.07 Ba	5.10 ± 0.16 Ca
MA2	387.83 ± 10.35 Ba	388.33 ± 10.84 Ba	0.91 ± 0.05 Ba	5.71 ± 0.23 Bb
MA3	455.22 ± 7.65 Ab	455.36 ± 11.84 Ab	0.99 ± 0.03 Aa	7.86 ± 0.32 Aa
MB1	390.02 ± 10.77 Ca	392.17 ± 12.86 Ca	0.80 ± 0.05 Bb	3.87 ± 0.18 Cb
MB2	492.16 ± 13.7 Bc	493.75 ± 9.86 Bc	0.93 ± 0.06 Aa	5.53 ± 0.35 Bb
MB3	926.76 ± 12.52 Aa	926.75 ± 12.16 Ab	0.98 ± 0.06 A	7.61 ± 0.49 Aa
MC1	306.20 ± 11.52 Cb	307.0 ± 8.65 Cb	0.89 ± 0.05 Aa	4.20 ± 0.23 Cb
MC2	414.29 ± 11.32 Bb	414.5 ± 9.53 Ba	0.98 ± 0.07 Aa	7.00 ± 0.14 Ba
MC3	914.21 ± 12.52 Aa	914.66 ± 13.54 Aa	0.98 ± 0.08 Aa	7.88 ± 0.26 Aa

Note: Different uppercase letters indicate significant differences between composting phases within the same treatment, while different lowercase letters indicate significant differences between treatments at the same phase (*p* < 0.05).

## Data Availability

Data are contained within the article.
